# Decomposing the effects of context valence and feedback information on speed and accuracy during reinforcement learning: a meta-analytical approach using diffusion decision modeling

**DOI:** 10.3758/s13415-019-00723-1

**Published:** 2019-06-07

**Authors:** Laura Fontanesi, Stefano Palminteri, Maël Lebreton

**Affiliations:** 1 Center of Economic Psychology, University of Basel, Basel, Switzerland; 2 Human Reinforcement Learning team, Université de Paris Sciences et Lettres, Paris, France; 3 Département d’études cognitives, Ecole Normale Supérieure, Paris, France; 4 Laboratoire de Neurosciences Cognitives et Computationnelles, Institut National de la Santé et Recherche Médicale, Paris, France; 5 Amsterdam Brain and Cognition, Universiteit van Amsterdam, Amsterdam, The Netherlands; 6 Center for Research in Experimental Economics and Political Decision-making, Amsterdam School of Economics, Universiteit van Amsterdam, Amsterdam, The Netherlands; 7 Neurology and Imaging of Cognition, Department of Basic Neurosciences, University of Geneva, Geneva, Switzerland; 8 Swiss Center for Affective Science, University of Geneva, Geneva, Switzerland

**Keywords:** Response time, Decision-making, Motivation, Reward, Decision diffusion model, Reinforcement learning

## Abstract

Reinforcement learning (RL) models describe how humans and animals learn by trial-and-error to select actions that maximize rewards and minimize punishments. Traditional RL models focus exclusively on choices, thereby ignoring the interactions between choice preference and response time (RT), or how these interactions are influenced by contextual factors. However, in the field of perceptual decision-making, such interactions have proven to be important to dissociate between different underlying cognitive processes. Here, we investigated such interactions to shed new light on overlooked differences between learning to seek rewards and learning to avoid losses. We leveraged behavioral data from four RL experiments, which feature manipulations of two factors: outcome valence (gains vs. losses) and feedback information (partial vs. complete feedback). A Bayesian meta-analysis revealed that these contextual factors differently affect RTs and accuracy: While valence only affects RTs, feedback information affects both RTs and accuracy. To dissociate between the latent cognitive processes, we jointly fitted choices and RTs across all experiments with a Bayesian, hierarchical diffusion decision model (DDM). We found that the feedback manipulation affected drift rate, threshold, and non-decision time, suggesting that it was not a mere difficulty effect. Moreover, valence affected non-decision time and threshold, suggesting a motor inhibition in punishing contexts. To better understand the learning dynamics, we finally fitted a combination of RL and DDM (RLDDM). We found that while the threshold was modulated by trial-specific decision conflict, the non-decision time was modulated by the learned context valence. Overall, our results illustrate the benefits of jointly modeling RTs and choice data during RL, to reveal subtle mechanistic differences underlying decisions in different learning contexts.

**Electronic supplementary material:**

The online version of this article (10.3758/s13415-019-00723-1) contains supplementary material, which is available to authorized users.

## Introduction

In cognitive psychology, the sequential sampling modeling (SSM) framework has enabled the development of models that jointly account for choice accuracy and response time (RT) data in two-alternative forced choice tasks (Gold & Shadlen, 2007; Bogacz et al., 2006; Smith & Ratcliff, 2004; Ratcliff & Smith, 2004). In this framework, it is assumed that, when evaluating two choice options, evidence in favor of one over the other alternative(s) is accumulated over time and a response is initiated when this evidence reaches a decision threshold. The crucial advantage of applying these models to empirical data is that they can help decompose the correlations between RTs and accuracy into meaningful psychological concepts. On the one hand, speed and accuracy can be positively correlated: e.g., when faced with easy decisions, people tend to give more correct and faster responses compared to when facing difficult decisions (Ratcliff & Rouder, 1998). This effect is captured in SSMs by higher rates of evidence accumulation. On the other hand, speed and accuracy can also be negatively correlated: e.g., when asked to make speedy decisions, people tend to be less accurate (Ratcliff & Rouder, 1998). This phenomenon is referred to as the speed–accuracy tradeoff (Heitz, 2008; Luce, 1986) and is explained within the SSM framework by a decrease in the decision threshold and interpreted as reduced cautiousness. Finally, speed and accuracy can also be uncorrelated: e.g., people can differ in how fast or slow they respond, without being more or less accurate (Ratcliff et al., 2003). These differences are captured in SSMs by the non-decision time parameter, which represents motor processes necessary for the execution of actions as well as time needed for stimulus encoding. Therefore, SSMs have provided a mechanistic explanation of these three different correlation patterns of RTs and accuracy and have been successfully applied in various psychological domains: from perceptual, to social, to economic decision-making, as well as in memory and language research (Ratcliff et al., 2016).

Research in reinforcement learning (RL) aims at characterizing the processes through which agents learn, by trial-and-error, to select actions that maximize the occurrence of rewards and minimize the occurrence of punishments (Sutton & Barto, 1998). A century-long experimental investigation of RL processes in human and non-human animals has shown that learning is accompanied by a simultaneous increase of the frequency of the selection of the most advantageous action and by a decrease of the time necessary to select this action (Pavlov, 1927; Skinner, 1938; Thorndike, 1911).

However, traditional computational RL models only account for choices and do not consider RTs (but see the recent work of Frank et al., 2015; Pedersen et al., 2017; Fontanesi et al., 2019). Therefore, how contextual factors in RL paradigms impact the relation between RTs and accuracy is still relatively poorly understood (Summerfield & Tsetsos, 2012).

In a series of recent studies, Palminteri and colleagues (Palminteri et al., 2015, 2016, 2017) developed an RL paradigm where they orthogonally manipulated two important contextual factors: feedback information and outcome valence. Feedback information was modulated by showing (i.e., complete feedback) or not showing (i.e., partial feedback) the outcome associated with the unchosen option. Outcome valence was modulated by reversing the sign of the outcome (i.e., gains vs. losses), which directly impacted the goal of learning: reward-seeking vs. punishment-avoidance. Independent analyses reported in the aforementioned studies consistently show that: First, and as expected, participants display a higher accuracy in complete feedback contexts, where more information is available to learn the value of options; second, participants learn equally well to seek rewards and to avoid punishments. This second finding is more surprising because losses have been demonstrated to have a greater psychological impact than gains—a phenomenon called loss aversion in behavioral economics (Kahneman & Tversky, 1979). Hence, one could expect that learning would be quicker in the loss contexts. Importantly though, RTs in the same task follow a different pattern: Participants are slower in the punishments contexts and in partial-feedback contexts. Therefore, despite apparent similarities in the choice pattern, we hypothesized that hidden asymmetries might exist between learning to seek reward, and learning to avoid losses.

Sequential sampling models (SSMs) can be used to investigate how different components of the decision process underpin the behavioral patterns observed in previous studies (Smith & Ratcliff, 2004). In the present paper, we first re-assess the effects of the contextual factors on RTs and accuracy using a meta-analytical approach involving data from four behavioral experiments employing the same RL paradigm. In a second step, we moved to the SSM framework and used a hierarchical Bayesian version of the standard diffusion decision model (DDM, Ratcliff 1978) to assess the effects of the contextual factors (i.e., feedback information and valence) on the model’s parameters (i.e., drift rate, threshold, and non-decision time). We found that the rate of evidence accumulation was higher in full-feedback compared to partial-feedback contexts, cautiousness was the lowest in the gain domain when the feedback information was partial, and the non-decision time increased in the loss domain as well as when the feedback was partial. While this first set of analyses confirms that the decision processes used in learning to seek rewards and learning to avoid losses might differ, the factorial DDM analyses do not take into account the sequential nature of reinforcement learning data and the trial-by-trial evolution of the underlying latent variables. To overcome these limitations, we fit a combination of the RELATIVE (Palminteri et al., 2015) model and the DDM, using an approach similar to Pedersen et al., (2017) and Fontanesi et al., (2019). Briefly, the RELATIVE model is a context-dependent reinforcement-learning model which efficiently accounts for the similar performances observed in gains and loss contexts, by using context value (i.e., an approximation of the overall value of a pair of choice options) as a reference point to compute prediction errors (Palminteri et al., 2015).

In line with previous findings (Fontanesi et al. 2019), we found that, in each trial, the difference in learned values determines the accumulation-rate, and the learned conflict increases the threshold. Most importantly, we also report for the first time that the learned contextual value decreases the non-decision time, thereby accounting for the slower RTs observed in loss contexts. Altogether, our results illustrate how RTs can be used to provide valuable information about the decision processes in instrumental learning paradigms. In particular, effects similar to the valence effects might be overlooked when considering choice data alone, thus providing a limited view of the decision processes at play.

## Methods

### Participants

We analyzed data from four behavioral experiments, realized in three different research centers in France and UK (final *N* = 89; Table 1). The local ethical committees approved the studies and participants provided written informed consent; see the original publications for additional details (Palminteri et al., 2015; Salvador et al., 2017).

**Table 1:** Participants

	Experiment 1	Experiment 2	Experiment 3	Experiment 4
Sample size	20	25	20	24
Mean age	25.4	23.9	32.4	22.2
Percentage males	55	36	55	38
Response window (s)	3	3	3	1.5
N sessions	2	3	3	2
N trials per session	80	96	96	80
Center	Paris - ENS	Paris - ENS	Paris - ICM	London- UCL
Source	Pilot for	Pilot for	Controls	Controls
Reference	Palminteri et al., (2015)	Palminteri et al., (2015)	Salvador et al., (2017)	Palminteri et al., (2016)

Note. Demographics, task characteristics, and investigation centers of the four experiments (N: sample size, ENS: École Normale Supérieure; ICM: Institut du Cerveau et de la Moëlle; UCL: University College London)

### Task

**Fig. 1:**
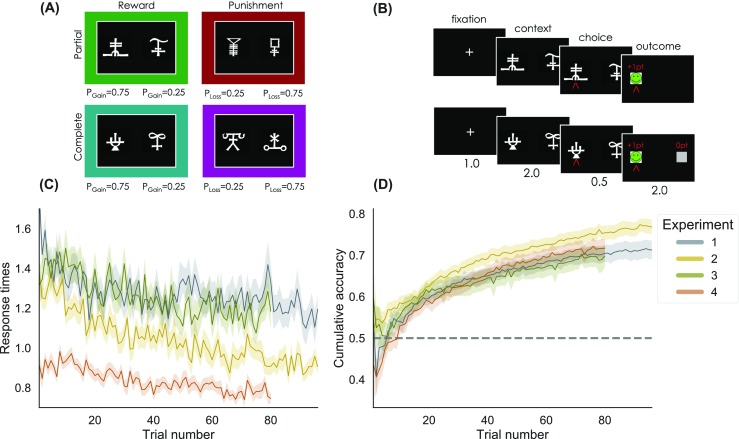
Task factors and learning curves. **a** The learning task 2×2 factorial design. Different symbols were used as cues in each context, and symbol to context attribution was randomized across participants. The *colored frames* are purely illustrative and represent each of the four context conditions throughout all figures. “Reward”: gain domain; “Punishment”: loss domain; “Partial”: only feedback of the chosen option is provided; “Complete”: both feedback of chosen and unchosen options are provided; *P*_*G*_*ain*= probability of gaining 1 point; *P*_*L*_*oss*= probability of losing 1 point. **b** Time course of example trials in the reward-partial (*top*) and reward-complete (*bottom*) conditions. Stimuli durations are given in seconds. **c** Average response times during learning. **d** Cumulative accuracy during learning. *Shaded areas* in **c** and **d** represent the 95% Bayesian credible intervals. The *horizontal dotted line* in **d** indicates chance level

Participants performed a probabilistic instrumental learning task designed to manipulate both feedback valence (reward vs. punishment) and feedback information (partial vs. complete) using a 2 × 2 factorial design (Fig. 1a). Participants had to choose one of two abstract cues (letters from the agathodaimon font). Each trial (Fig. 1B) started with a fixation cross, followed by presentation of the cues during which participants indicated their choice. After the choice window (either 3 or 1.5 s, depending on the experiment), a red arrow highlighted the chosen option. Then, the outcome was revealed, and participants moved to the following trial. In each session, there were eight different cues, divided into four fixed pairs, corresponding to four choice contexts: reward-partial, reward-complete, punishment-partial, and punishment-complete. In reward contexts, the best cue had 75% probability of yielding a reward (points or money) and 25% probability of yielding nothing; while the worst cue, on the other hand, had 25% probability of yielding a reward and 75% probability of yielding nothing. In punishment contexts, the best cue had 25% probability of yielding a loss and 75% probability of yielding nothing, while the worst cue had 75% probability of yielding a loss and 25% probability of yielding nothing. In partial feedback contexts, participants were presented with only the outcome of the chosen cue, while in complete feedback contexts they were presented with the outcomes of both the chosen and forgone cues. The number of trials per context, the number of sessions, and the timing slightly differed across experiments (see Table 1).

### Dependent variables

Our main dependent variables were the correct choice rate (accuracy) and RTs. A correct response is defined as a choice directed toward the best (reward maximizing or punishment minimizing) cue of a pair. The RT is defined as the time between the presentation of the options and the button press.

### Bayesian analysis of the variance

Accuracy and RTs were analyzed in two independent ANOVAs, which modeled the main effects of—and the interaction between—the experimental manipulations (i.e., valence and feedback information). We adopted a Bayesian mixed model meta-analysis approach, where the different experiments could be modeled as fixed effects (Singmann et al., 2014). By doing so, we could test whether, across the four experiments, mean accuracy and RTs differed and whether the learning contexts were similar across the experiments.

This approach entails a comparison of different Bayesian models using Bayes factors (BFs) (Kass and Raftery, 1995; Wagenmakers, 2007) in a two-step procedure. First, we assessed whether the experiments should be treated as fixed effects by comparing such a model to a model with only the random effect of participants. The winning model was then used as a baseline model in the second step, where we assessed which combinations of fixed-effects and interactions gave the most parsimonious, but complete account of the data. Once we identified the best model, we inspected the estimated posterior distribution of its main effects and interactions (see Appendix A). The models were all fit using the R package BayesFactor (Morey et al., 2015) and adapted code previously provided by Singmann et al., (2014).

### Diffusion decision model architecture

The DDM (Ratcliff 1978, 1998) assumes that, when deciding between two alternatives, evidence in favor of one relative to the other is accumulated in time, according to the following differential equation:

1 $$  dx = \mathcal{N}(v \cdot dt, c \cdot \sqrt{dt}), x_{0} = a/2 $$

where *dx* is the change in the accumulated evidence in the time interval *dt*, *v* is the mean accumulated evidence across the time intervals, and *c* is the noise constant, usually fixed to 1 1. A decision is executed when enough relative evidence in favor of an alternative has been collected, which is when *x* is either lower than 0 or higher than the decision threshold *a*. When the decision is unbiased (i.e., there is equal initial evidence in favor of both options), then the evidence accumulation starts from half the threshold *a*. In the experiments that were considered in the present study, the upper boundary corresponded to the correct option (i.e., the option with the highest mean payoff) and the lower boundary corresponded to the incorrect option (i.e., the option with the lowest mean payoff) within a context. Because these options were randomly assigned to the right and left sides of the screen, we assumed that decisions were always unbiased, and coded responses as correct and incorrect.

Therefore, the execution time and probability of choosing the option with the highest payoff depended on three main parameters. The first is the decision threshold *a*: lower thresholds lead to faster but less accurate decisions, while higher thresholds lead to slower but more accurate decisions. The threshold is usually interpreted as response caution, with higher thresholds corresponding to higher cautiousness. The second parameter is the drift rate *v*, which is the amount of evidence accumulated per unit of time. This can reflect the difficulty of the decision problem, as well as participants’ efficiency in the task: higher drift rates lead to faster as well as more accurate responses. The third parameter that we take into account is referred to as *non-decision time* (NDT), and reflects the processes that influence the decision time, but does not pertain to evidence accumulation per se, such as motor and stimuli encoding processes. The non-decision time therefore affects RTs without affecting accuracy.

### Diffusion decision model fitting

For each of the DDM parameters (i.e., *v*, *a*, and NDT), we fitted an intercept and three slopes, corresponding to the two main effects–valence and feedback information—and their interaction. This allowed us to test the effects of the experimental manipulations on the model parameters. To account for all levels of variability, we used a three-level version of the hierarchical Bayesian DDM, where the first level corresponds to the participants, the second corresponds to the experiments, and the third corresponds to the whole dataset, thus mimicking the meta-analysis approach described for the Bayesian analysis of the variance.

To fit the Bayesian DDM and estimate its joint posterior distribution, we used *stan*, a probabilistic programming language for Bayesian parameter estimation (Carpenter et al., 2017). In particular, we ran four independent chains with 10,000 samples each, and discarded the first half of each chain. To test for convergence, we checked that the $\hat {R}$ statistic (Gelman and Rubin, 1992)—a measure of convergence across chains—was lower than 1.01 for all parameters. See the Appendix B for details about the prior distributions. To test the reliability of the parameter estimates, we performed parameter recovery on a simulated dataset (Palminteri et al., 2017) (see the Appendix C).

Finally, to assess the model fit of the DDM, we computed the posterior predictive distributions (Gelman et al., 1996) for mean accuracy and RTs, as well as for RT quantiles (separately for correct and incorrect responses; Fig. B2).

### Reinforcement learning architecture

To capture the trial-by-trial dynamics due to learning-by-feedback, we fitted a combination of the “RELATIVE” model, proposed by Palminteri et al., (2015), and of the DDM. The RELATIVE model is based on a simple Q-learning model (Sutton and Barto, 1998), but allows separate learning-rate parameters for outcomes of chosen and forgone options, and includes a contextual module, so that option values are updated relative to the learned value of the choice context.

In the RELATIVE model, at each trial *t*, the option values *Q* in the current context *s* are updated with the Rescorla–Wagner rule (Rescorla & Wagner, 1972):

2 $$ \begin{array}{@{}rcl@{}} Q_{c,s,t} = Q_{c,s,t-1} + \alpha_{c} \cdot \delta_{c}&& \\ Q_{u,s,t} = Q_{u,s,t-1} + \alpha_{u} \cdot \delta_{u}&& \end{array} $$

where *α*_*c*_ is the learning rate for the chosen option *Q*_*c*_—updated in both partial and complete feedback contexts—and *α*_*u*_ the learning rate for the unchosen option *Q*_*u*_—updated only in complete feedback contexts. *δ*_*c*_ and *δ*_*u*_ are prediction error terms, calculated as follows:

3 $$ \begin{array}{@{}rcl@{}} \delta_{c} = R_{c,s,t} -V_{s,t-1} -Q_{c,s,t-1} &&\\ \delta_{u} = R_{u,s,t} -V_{s,t-1} -Q_{u,s,t-1}&& \end{array} $$

*V*_*s*_ represents the context value that is used as the reference point for the updating of option values in a particular context, and *R* is the feedback received in a trial. Context value is also learned via a delta rule:

4 $$ V_{s,t} = V_{s,t-1} + \alpha_{V} \cdot \delta_{V} $$

where *α*_*V*_ is the learning rate of context value and *δ*_*V*_ is a prediction error term. In complete feedback contexts:

5 $$ \delta_{V} = \frac{(R_{c,s,t}+R_{u,s,t})}{2} -V_{s,t-1} $$

In partial feedback contexts, since *R*_*c*,*s*,*t*_ is not provided, its value is replaced by its expected value *Q*_*u*,*s*,*t*_, hence:

6 $$ \delta_{V} = \frac{(R_{c,s,t}+Q_{u,s,t})}{2} -V_{s,t-1} $$

The decision rule was implemented as in Eq. 1 (i.e., according the diffusion decision model). This approach, of tightly linking RL models to the DDM, was previously proposed by Pedersen et al., (2017) and Fontanesi et al., (2019). In this way, we could test specific hypotheses of how the latent learning variables affect the decision components.

The first hypothesis is that the drift rate is determined by the trial-by-trial difference in the learned values, Δ*Q*_*t*_. To test this hypothesis, we defined the drift rate in each trial *v*_*t*_ as:

7 $$ \begin{array}{@{}rcl@{}} {\Delta} Q_{t}=(Q_{\text{cor},t} - Q_{\text{inc},t}) \end{array} $$

8 $$ \begin{array}{@{}rcl@{}} v_{t} = v_{\text{coeff}} \cdot {\Delta} Q_{t} \end{array} $$

where *v*_coeff_ is the drift-rate coefficient and *Q*_cor,*t*_ and *Q*_inc,*t*_ are the learned expectations of the correct and incorrect options in a trial. This hypothesis was also tested and confirmed in previous instances of RLDDM (Pedersen et al., 2017; Fontanesi et al., 2019). This mechanism could help to explain the feedback effect on both accuracy and RTs.

The second hypothesis is that the threshold is modulated by the trial-by-trial conflict, defined as the inverse of the absolute difference between the Q values of the options 1/(|Δ*Q*_*t*_| + 1):

9 $$  a_{t} = a_{\text{int}} \cdot \{1 + a_{\text{coeff}} \cdot [1/(|{\Delta} Q_{t}|+1) - 1]\} $$

where *a*_int_ is the threshold intercept and *a*_coeff_ is the threshold coefficient, where 0 ≤ *a*_coeff_ ≤ 1. Since conflict is bounded between 0 and 1, the more the threshold coefficient approaches 1, the more the threshold intercept is discounted by lower conflict. When the threshold coefficient is 0, conflict does not affect the threshold intercept. This parameterization also prevents the threshold from being negative. This hypothesis is in line with previous models that proposed modulations of the threshold parameters due to conflict (Frank et al., 2015; Cavanagh et al., 2014), although it has not been tested yet in a simultaneous RL and DDM fitting. This mechanism could help to explain a possible interaction of feedback and valence on RTs.

The third and last hypothesis is that the non-decision time is modulated by the trial-by-trial contextual valence *V*_*t*_, defined in Eq. 4:

10 $$  NDT_{t} = exp(NDT_{\text{int}} + NDT_{\text{coeff}} \cdot V_{t}) $$

where *NDT*_int_ is the threshold intercept and *NDT*_coeff_ is the threshold coefficient. The non-decision time is exponentially transformed to ensure that it is always positive. A possible non-decision time modulation of valence was previously proposed by Ratcliff and Frank (2012) but was never tested in a simultaneous RL and DDM fitting.

### Reinforcement learning model fitting

We fitted a hierarchical Bayesian version of the RLDDM simultaneously on the choice and response times data, separately for each experiment. See the Appendix D for details about the prior distributions. The RL model was coded and fitted using *stan*, using the same parameters and procedures described above for DDM fitting.

To assess the model fit of the RLDDM, we computed the posterior predictive distributions (Gelman et al., 1996) for mean accuracy and RTs, separately by bins of trials and learning context, and for experiment (see Fig. D2).

### Statistical reporting

In all analyses (i.e., ANOVA, linear mixed-effect regression and DDM), we report the estimated Bayesian credible interval (BCI) of the posterior distributions of the parameters of interest, computed as the 95% central interval of the distributions.

In all analyses, valence was coded as 0 for reward and 1 for punishment, and feedback was coded as 0 for partial and 1 for complete. Intercepts therefore correspond to the reward-partial context. The interaction was obtained by multiplying valence and feedback.

## Results

### Bayesian analysis of the variance

**Fig. 2:**
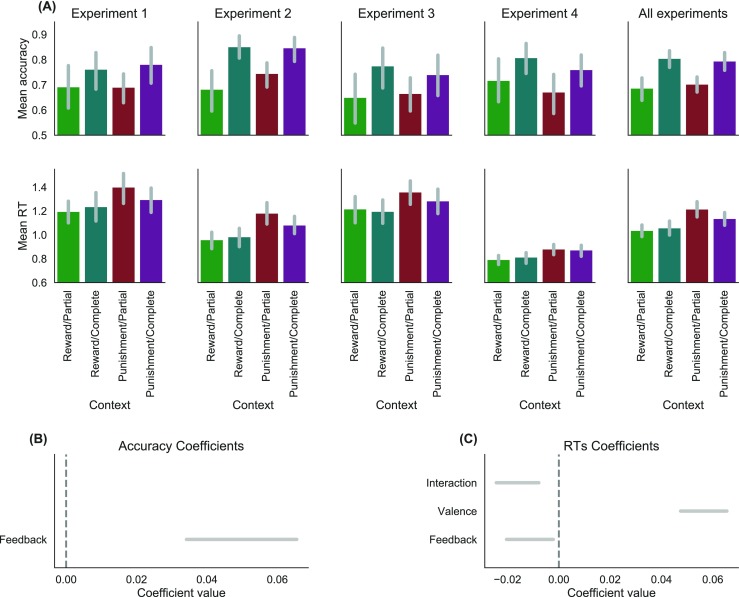
Performance and behavioral effects across learning. **a** Summary of the behavioral performance. Mean accuracy (*top*) and response times in seconds (*bottom*) are plotted, separately for experiments and conditions, as well as across experiments (*right column*). The *bars* represent 95% confidence intervals. *Bottom row*: 95% Bayesian credible intervals of the posterior distributions of the feedback, valence, and feedback–valence interaction effects on accuracy (**b**) and RTs (**c**) of the preferred models in the ANOVA model comparison analyses

We assessed the effects of outcome valence and feedback information on learning performance (i.e., mean accuracy and RTs, Fig. 2A), using a Bayesian mixed model meta-analysis approach (see Methods).

For the accuracy, our approach favored a model with (1) a single main effect accounting for feedback information, (2) no main effect of the experiment, (3) no interactions between experiment and experimental manipulations (M3 in Table A1). These results indicate that only feedback and not valence had an effect on accuracy, and that this effect had a similar size across the experiments. The model parameters confirmed that accuracy was higher in the complete feedback information contexts (BCI_Feedback_ = [.03 – .06]) (see Fig. 2 B).

For the RTs, our approach favored a model that includes (1) both main effects of valence and feedback information as well as their interaction, (2) a main effect of the experiment, (3) and no interaction between experiment and experimental manipulations (M5 in Table A2). These results indicate that both valence and feedback information, as well as their interaction, had an effect on RTs, in a similar way across the experiments. The main effect of the experiment indicates that participants had different mean RTs across the experiments. The model parameters revealed that participants were slower in the loss domain (BCI_Valence_ = [.05 – .07]) and faster in the complete feedback contexts (BCI_Feedback_ = [-.020 – -.002]). In addition, the effect of valence was weaker in the complete feedback contexts (BCI_Interaction_ = [-.02 – -.01]).

### Diffusion decision model analyses

Although the two ANOVAs depict a picture of the effect of different learning contexts on both RTs and accuracy that is consistent across the experiments, they do not model the interactions between accuracy and RTs. To decompose the simultaneous effects of contextual effects on RTs and accuracy, we therefore fitted a three-level hierarchical Bayesian version of the DDM to the data of all four experiments.

**Fig. 3:**
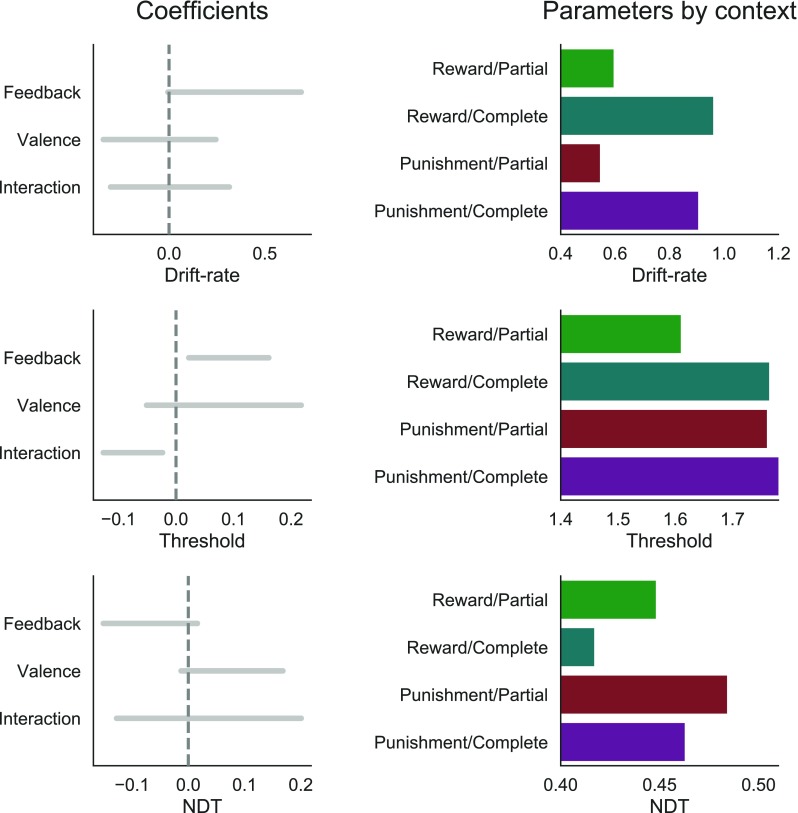
Estimated diffusion decision model (DDM) parameters. *Left column*: 95% Bayesian credible intervals of the estimated posterior distributions of the effects of the experimental manipulations (i.e., feedback information, outcome valence, and their interaction) on the DDM parameter coefficients at the dataset level. *Right column*: estimated mean parameters at the dataset level, separately by context

The increase in accuracy and speed in the complete feedback contexts was captured by an effect on all three DDM parameters (Fig. 3): Providing participants with complete feedback increased the drift rate (BCI = [-.01 – .69]), increased the threshold (BCI = [.02 – .16]), and decreased the non-decision time (BCI = [-.151 – .016]). Compared to the gain domain, decisions in the loss domain showed higher threshold (BCI = [-.05 – .22]) and non-decision time (BCI = [-.013 – .168]). Valence did not affect the drift rate (BCI = [-.34 – .24]). Importantly, the valence effect on the threshold was different across the four experiments, with a stronger effect in experiment 3, and a weaker effect in experiment 4 (Fig. B1). This might be due to the higher time pressure in experiment 4. Yet, we found a negative interaction between feedback information and valence on the threshold (BCI = [-.13 – -.02]). A closer examination of the threshold parameter by context (Fig. 3, right column) revealed that the threshold was particularly low in the reward-partial condition. There was no interaction effect on the non-decision time (BCI = [-.128 – .201]), nor on the drift rate (BCI = [-.31 – .31]). Finally, while the drift-rate intercepts were similar across experiments, threshold and non-decision time varied across experiments, with a lower threshold in experiment 4, and a higher non-decision time in experiment 3 (see Fig. B1, top row). In Fig. B1 we report the posterior distributions of the group parameters separately for experiments as well as for the overall dataset.

### Reinforcement learning model analyses

A limit of both the ANOVAs and of the DDM analyses is that they do not take into account the sequential nature of the data and the trial-by-trial evolution of the underlying latent variables. To overcome this limitation, we fit a combination of a RL model and the DDM that allows us to test the relationship between latent learning and decision processes. Regarding the RL model implementation, we chose the RELATIVE model, first proposed by Palminteri et al., (2015). The crucial ideal behind the model is that the agent learns values on a relative (i.e., context-dependent) scale. To achieve context-dependence, the model tracks, in addition to action values *Q*, the context values *V*.

In particular, we were interested in linking three latent variables of the RELATIVE model with the drift rate, threshold, and non-decision time parameters of the DDM. Based on previous behavioral findings, we focused on the learned difference between the correct and incorrect options’ values Δ*Q*_*t*_, on the learned decision conflict 1/(|Δ*Q*_*t*_| + 1), and on the context value *V*_*t*_, separately by experiment.

**Fig. 4:**
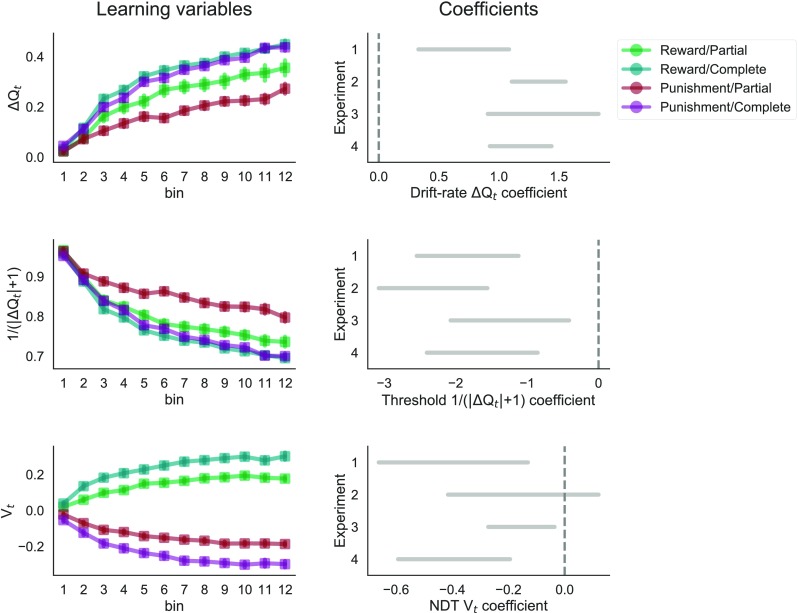
Estimated reinforcement learning diffusion decision model (RLDDM) parameters. *Left column*: development of the latent learning variables (i.e., difference in learned Q values, trial-conflict, contextual value) throughout learning, as predicted by the RLDDM (by context, and across experiments). *Right column*: 95% Bayesian credible intervals of the estimated posterior distributions of the effects of the latent learning variables on the DDM parameters (respectively, drift rate, threshold, and non-decision time) at the experiment level

The Δ*Q*_*t*_ started at zero and increased throughout learning in all learning contexts, the more so in complete as opposed to partial feedback contexts (Fig. 4, top left), as predicted by the learning rules of the RELATIVE model. The drift-rate coefficients for Δ*Q*_*t*_ were positive in all four experiments (BCI = [0.33 – 1.09], [1.11 – 1.56], [0.91 – 1.83], [0.93 – 1.44]), meaning that the drift rate was positively modulated by the learned difference in values (Fig. 4, top right).

The conflict 1/(|Δ*Q*_*t*_| + 1) tended to decrease throughout learning in all learning contexts, the more so in complete as opposed to partial feedback contexts (Fig. 4, middle left). The threshold coefficients for conflict were negative in all experiments (BCI = [-2.54 – -1.12], [-3.08 – -1.56], [-2.07 – -0.41], [-2.40 – -0.85]), meaning that the threshold was negatively modulated by the learned conflict (Fig. 4, middle right).

Finally, contextual value *V*_*t*_ tended to increase in rewarding and to decrease in punishing contexts throughout learning, (Fig 4, bottom left). The non-decision time coefficients for *V*_*t*_ were negative in all but one experiment (BCI = [-0.66 – -0.13], [-0.42 – 0.12], [-0.27 – -0.04], [-0.59 – -0.19]), meaning that the non-decision time was negatively modulated by the learned contextual value (Fig. 4, bottom right).

The complete set of group level parameter posterior distributions of the RLDDM can be seen in Fig. D1. Posterior predictive checks indicated that the RLDDM also showed a good fit to the data, as can be seen in Fig. D2, for both mean RTs and accuracy, across experiments, learning, and contexts.

## Discussion

In the present study, we looked at how different RL contexts (i.e., partial vs. full feedback, and gains vs. losses) affect accuracy and RTs. To do so, we used different methods and a relatively large dataset, composed of four separate experiments carried out in different centers.

First, we used a meta-analytic Bayesian approach to the analysis of variance of accuracy and RTs. Replicating previous reports (Palminteri et al.,, 2016; Salvador et al., 2017), we showed that participants were slower in the loss (as compared to the gain) domain, and that they were more accurate and faster when complete (as compared to partial) feedback was provided. Interestingly, the similar accuracy observed in the gain and loss domains is at odds with the notion of loss aversion (Kahneman and Tversky, 1979): If in our task “losses loomed greater than gains”, we would expect higher accuracy in the loss domain. However, by inspecting the RTs, we found that losses made participants slower, showing the importance of simultaneously considering complementary aspects of performance (i.e., choice and response time) to build psychological theories.

Because the ANOVAs do not allow to inspect RTs and accuracy simultaneously, and to better understand this effect on RTs (as well as the interaction between valence and feedback information on RTs), we turned to the SSM framework and fitted the DDM simultaneously to accuracy and RTs across the four experiments. Previous studies that applied the SSM framework to value-based decision-making have shown how the difficulty effect can be captured by a decrease in the mean accumulation rate (Milosavljevic et al., 2010; Cavanagh et al., 2014; Frank et al., 2015; Krajbich et al., 2010). However, previous studies investigating the valence effect have given mixed interpretations (Ratcliff & Frank, 2012; Cavanagh et al., 2014). We found that the effect of feedback information (i.e., higher accuracy and speed in the complete contexts) appeared to be driven by an increase of the drift rate and of the threshold parameters, and by a decrease of the non-decision time in the complete compared to partial conditions (thus transcending mere difficulty effects). On the other hand, valence had a main effect on the non-decision time and threshold, and there was an interaction of feedback and valence on the threshold (with lowest threshold in the reward-partial condition). The effect of valence on threshold (higher thresholds in the loss domain) was not consistent across experiments, and it was higher in experiments with less time pressure.

**Fig. 5:**
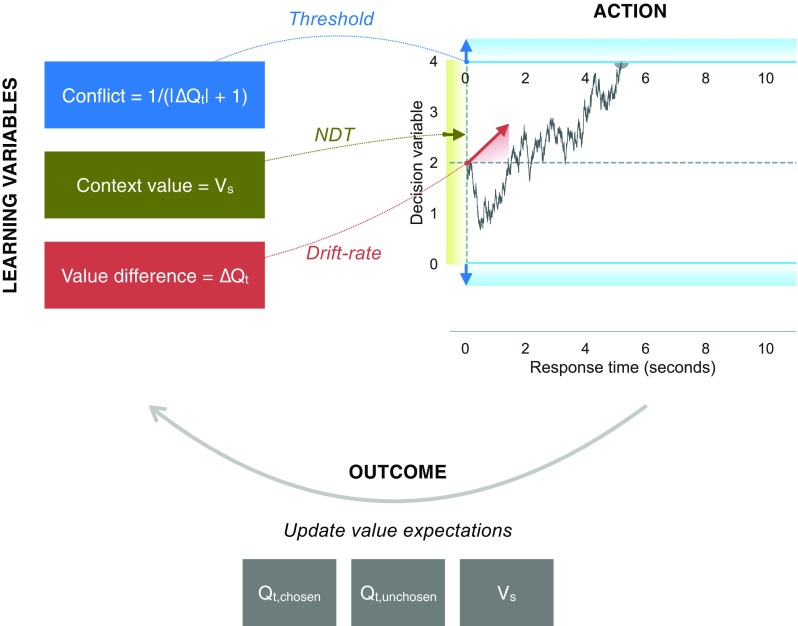
Illustration of the reinforcement learning diffusion decision model (RLDDM). In each trial, the learned conflict, context value, and value difference modulate, respectively, the decision threshold, the non-decision time, and the drift rate of the drift diffusion model. After experiencing the actions’ outcomes, the value expectations are updated following the RELATIVE model learning rules

These results were further supported by the RLDDM analyses (see Fig. 5): The learned context values derived from the RELATIVE model (Palminteri et al., 2015) affected RTs on a trial-by-trial base by modulating the non-decision time parameter of the DDM (in all but one experiments). In the RELATIVE model, context value is used as reference point in a particular context to update the Q values in each trial. (Palminteri et al., 2015) showed that including context value in the RELATIVE model improves the model fit to choice data (by comparing the RELATIVE model to similar RL models without contextual learning). Here we showed that context value can also be used to explain RTs data. Because the RELATIVE model decision rule (i.e., the softmax choice rule) does not predict RTs, this relationship had not been investigated so far. In addition to the context values, other psychologically relevant quantities can be derived from the RELATIVE model latent variables. Here, we derived conflict in each trial (Cavanagh et al., 2014) as the inverse of the absolute difference of the learned values of the available options. In line with previous studies (e.g., Cavanagh et al., 2014; Frank et al.,^2015^), we show that conflict modulates the decision threshold parameter of the DDM: participants were more cautious in higher conflict trials. Finally, confirming previous RLDDM approaches (Fontanesi et al., 2019; Pedersen et al., 2017), the learned differences in values determined the drift rate on a trial-by-trial basis.

While drift-rate difficulty effects have been documented in both economic and perceptual decision- making (Milosavljevic et al., 2010; Krajbich et al., 2010; Ratcliff & Rouder, 1998), the decrease in threshold in partial feedback contexts may appear counter-intuitive at first glance, as less information, and therefore higher uncertainty, could increase cautiousness. Moreover, previous studies have found that higher difficulty also leads to an increase in the threshold (e.g., Frank et al., 2015). Yet, a possible psychological interpretation for this effect is that the outcomes corresponding to the unchosen options are known to elicit regret, which can increase cautiousness in decision-making (Zeelenberg, 1999; Shenhav et al., 2014). This can thus explain the interaction effect on the threshold, since regret should be the lowest in the reward-partial condition.

The two effects on the non-decision time (of both feedback and valence) are less standard: non-decision time effects are not very common in the SSM literature, as they are thought to reflect stimulus encoding or purely motor processes (Ratcliff & Rouder, 1998). Alternative accounts of the RT slowing in the loss domain typically predict higher accuracy for losses. In decision field theory (Busemeyer & Townsend, 1993), for example, choices in the loss domain are characterized by a slowing down of the evidence accumulation process dependent on the distance from the decision threshold, thus causing slower and more accurate responses. SSMs that assume a race between the evidence accumulation of competing options (e.g., Brown & Heathcote 2008), also predict differences in accuracy. Finally, Hunt et al., (2012) proposed a biophysically plausible network model that predicted slower decisions when choosing between options with overall lower value. Since all these models concomitantly predict response time slowing and an increase in accuracy, they are not perfectly suited to explain the phenomena we observed.

A possible explanation of the increase in non-decision time in the loss domain is that negative valence contexts might induce motor inhibition, similarly to a Pavlovian bias (Boureau and Dayan, 2011; Huys et al., 2011). This effect is also similar to the modulating function of the subthalamic nucleus in the basal ganglia circuit, which causes a “hold your horses” response (Frank, 2006) in the presence of conflict. This would explain why responses could be delayed without affecting accuracy.

A competing explanation might link the slowing down in the presence of losses to the loss attention framework (Yechiam & Hochman, 2013), i.e., the idea that losses receive more attention. However, increased attention has been previously linked to increases in the drift rate and threshold parameters, and not in the non-decision time, since higher attention is typically accompanied by higher accuracy (Krajbich et al., 2010, 2012).

Finally, both effects of losses and partial feedback might not only be present in RTs, but also in meta-cognitive judgments like decision confidence. This idea is supported by a growing body of evidence showing how losses reduce confidence judgments in a variety of tasks (Lebreton et al., 2018, 2019).

In conclusion, RTs and accuracy are two behavioral manifestations of internal decision processes. These two variables provide complementary and equally important clues on the computations underpinning affective decision-making, and should be jointly considered in order to build a comprehensive account of goal-directed behavior.

## Electronic supplementary material

Below is the link to the electronic supplementary material.

(PDF 361 KB)

## Data Availability

https://osf.io/rvkd2/

## Footnotes

1 This is done to be able to identify the other parameters. One could decide to fix a different parameter, e.g., the decision threshold, to estimate this variable instead.
