# Supplementary material for: Decomposing the effects of context valence and feedback information on speed and accuracy during reinforcement learning: a meta-analytical approach using diffusion decision modeling
Source: Cogn Affect Behav Neurosci. 2019 Jun 7;19(3):490–502. doi: 10.3758/s13415-019-00723-1 (PMC6598978; doi:10.3758/s13415-019-00723-1)
Supplement: Supplementary file 1 — (PDF 361 KB) [file 13415_2019_723_MOESM1_ESM.pdf]

Decomposing the Effects of Context Valence and Feedback Information on Speed and Accuracy During Reinforcement Learning: A Meta-Analytical Approach Using Diffusion Decision Modeling – Supplementary material

Laura Fontanesi[1], Stefano Palminteri*[2,3,4], and Maël Lebreton*[5,6,7,8]

[1]Center of Economic Psychology, University of Basel, Basel, Switzerland
[2]Human Reinforcement Learning team, Université de Paris Sciences et Lettres, Paris, France
[3]Département d'études cognitives, Ecole Normale Supérieure, Paris, France
[4]Laboratoire de Neurosciences Cognitives et Computationnelles, Institut National de la Santé et Recherche Médicale, Paris, France
[5]Amsterdam Brain and Cognition (ABC), Universiteit van Amsterdam, Amsterdam, the Netherlands
[6]CREED, Amsterdam School of Economics (ASE), Universiteit van Amsterdam, Amsterdam, the Netherlands
[7]Neurology and Imaging of Cognition, Department of Basic Neurosciences, University of Geneva, Geneva, Switzerland
[8]Swiss Center for Affective Science, University of Geneva, Geneva, Switzerland

*Co-supervision.
Corresponding author: stefano.palminteri@gmail.com

Appendix A
Bayesian mixed model ANOVA

In this section, we detail the results of the two-step model-comparison approach that we used for the Bayesian Mixed Model ANOVA.

In a first step, to determine what would be the base model for the subsequent analyses, we compared two models: the first one (M0) included only participants as random effects and the second one (M1) included also experiment as fixed effect. In the case of accuracy, a model that does not include experiment as fixed effect was preferred (BF_{M0}/ BF_{M1}=3.6), indicating that mean accuracy was mostly stable across experiments. In the case of RTs, a model that includes experiment as fixed effect was preferred (BF_{M1}/ BF_{M0}=1.4e9), indicating that mean RTs differed across experiments.

In a second step, we tested different combinations of models in which we varied the possible interactions between experiment and experimental manipulations and the experimental manipulations themselves. In the case of accuracy, all models were tested against M0 of the previous step of the analyses, while, in the case of RTs, these were tested against M1 of the previous analyses. The results are summarized in Tables A1 and A2.

Finally, the two models with highest BF were compared to each other, to provide a simple assessment of the evidence in favor of the best model is. There was substantial evidence for the winning model in the ANOVA of accuracy analyses, M3, compared to its runner-up, M8 (BF_{M3}/ BF_{M8}=8.6). There was anecdotal evidence for the winning model in the ANOVA of RT analyses, M5, compared to its runner-up, M10 (BF_{M5}/ BF_{M10}=1.76).

Table A1

Bayes Factors of the ANOVA of accuracy.

Model	Random effects	Experiment interactions	Fixed Effects	log(BF)
M3	participant	None	Feedback	16.93
M8	participant	Valence	Feedback	14.79
M4	participant	None	Feedback + Valence	14.74
M13	participant	Feedback	Feedback	14.13
M5	participant	None	Feedback * Valence	13.17
M9	participant	Valence	Feedback + Valence	12.60
M18	participant	Feedback + Valence	Feedback	12.00
M14	participant	Feedback	Feedback + Valence	11.95
M10	participant	Valence	Feedback * Valence	11.03
M15	participant	Feedback	Feedback * Valence	10.38
M19	participant	Feedback + Valence	Feedback + Valence	9.81
M20	participant	Feedback + Valence	Feedback * Valence	8.25
M2	participant	None	Valence	-2.19
M6	participant	Valence	None	-2.33
M11	participant	Feedback	None	-2.33
M7	participant	Valence	Valence	-4.51
M12	participant	Feedback	Valence	-4.53
M16	participant	Feedback + Valence	None	-4.65
M17	participant	Feedback + Valence	Valence	-6.84

Note. The preferred model is marked with an asterisk.

Table A2

Bayes Factors of the ANOVA of response times.

Model	Random effects	Experiment interactions	Fixed Effects	log(BF)
M5	participant	None	Experiment + Feedback * Valence	62.37
M10	participant	Valence	Experiment + Feedback * Valence	61.81
M15	participant	Feedback	Experiment + Feedback * Valence	60.51
M20	participant	Feedback + Valence	Experiment + Feedback * Valence	59.99
M4	participant	None	Experiment + Feedback + Valence	57.38
M9	participant	Valence	Experiment + Feedback + Valence	56.66
M2	participant	None	Experiment + Valence	56.48
M7	participant	Valence	Experiment + Valence	55.69
M14	participant	Feedback	Experiment + Feedback + Valence	55.43
M19	participant	Feedback + Valence	Experiment + Feedback + Valence	54.74
M12	participant	Feedback	Experiment + Valence	54.34
M17	participant	Feedback + Valence	Experiment + Valence	53.58
M3	participant	None	Experiment + Feedback	-0.24
M6	participant	Valence	Experiment	-0.47
M8	participant	Valence	Experiment + Feedback	-0.66
M11	participant	Feedback	Experiment	-2.67
M13	participant	Feedback	Experiment + Feedback	-2.80
M16	participant	Feedback + Valence	Experiment	-3.12
M18	participant	Feedback + Valence	Experiment + Feedback	-3.19

Note. The preferred model is marked with an asterisk.

Appendix B
Diffusion decision model analyses

In this section, we report some details about the diffusion decision model analyses.

The following prior distributions were assumed for the parameter intercepts:

$$v_{\text{int}} \sim Cauchy(0,5) + z_i + z_j$$
$$a_{\text{int}} \sim Cauchy(0,5) + z_i + z_j$$
$$NDT_{\text{int}} \sim Cauchy(0,5) + z_i + z_j$$

where *Cauchy* is Cauchy distribution with parameters location and scale. The following prior distributions were assumed for the parameter coefficients (coefficients corresponding to the main and interactions effect were given the same priors):

$$v_{\text{coeff}} \sim Cauchy(0,5) + z_i + z_j$$
$$a_{\text{coeff}} \sim Cauchy(0,5) + z_i + z_j$$
$$NDT_{\text{coeff}} \sim Cauchy(0,5) + z_i + z_j$$

z_i and z_j respectively account for individual ($1 \leq i \leq 89$) and experiment ($1 \leq j \leq 4$) deviations from the group mean: z_i represents the deviation of a participant's parameter from that parameter mean in the experiment, while z_j represents the deviation of an experiment's parameter mean from the parameter means across the overall dataset. The following prior distributions were given to z_i and z_j:

$$z_i \sim \mathcal{N}(0, \sigma_j)$$
$$z_j \sim \mathcal{N}(0, \sigma)$$

σ_j ($1 \leq j \leq 4$) and σ respectively account for the within- and across-experiment variances, and have priors:

$$\sigma_j \sim HalfCauchy(0,5)$$
$$\sigma \sim HalfCauchy(0,5)$$

where $HalfCauchy$ is a strictly positive Cauchy distribution.

To constrain the threshold and non-decision time parameters to be positive, the exponentially transformed them at the trial level.

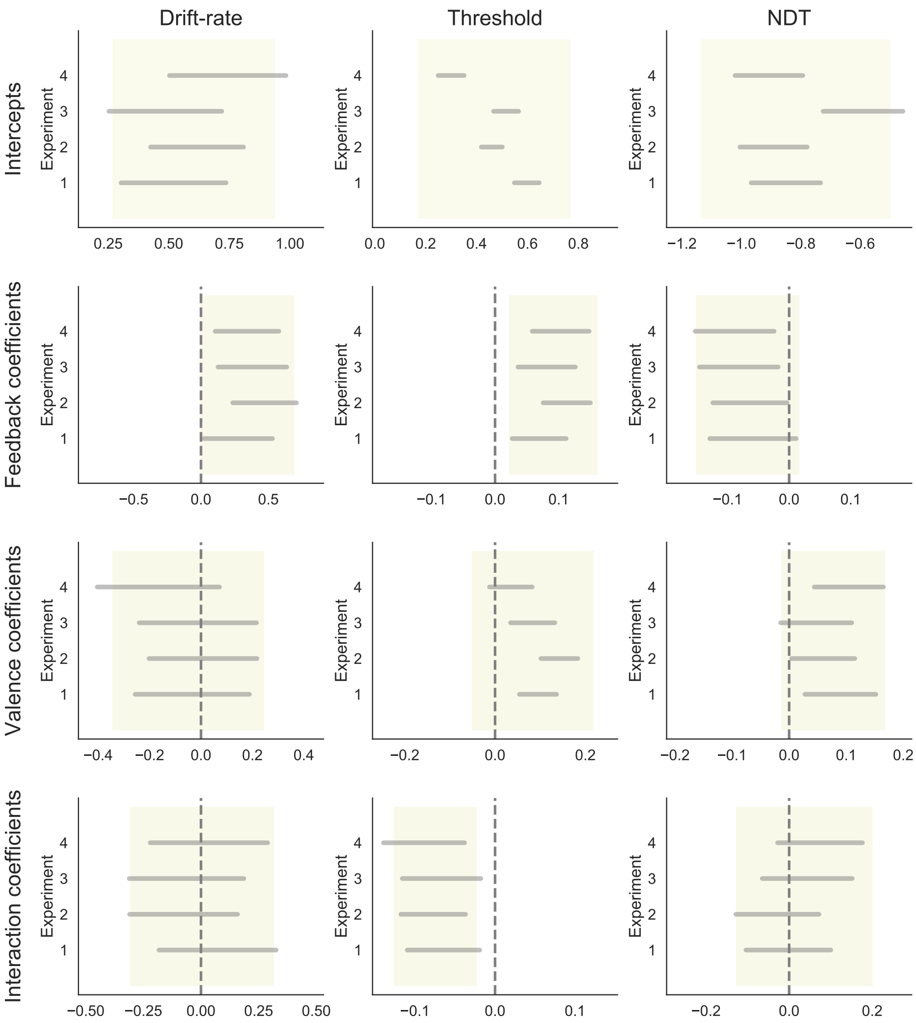

Figure B1. *Posterior distributions of the group parameters of the hierarchical diffusion decision model.* Posterior distributions of the DDM parameters across experiments (beige areas) and within experiments (grey lines). Because valence was coded as 0=reward/1=punishment, and feedback was coded as 0=partial/1=complete, and the interaction was the product of the two, intercepts (first row) correspond to the parameters in the reward-partial condition.

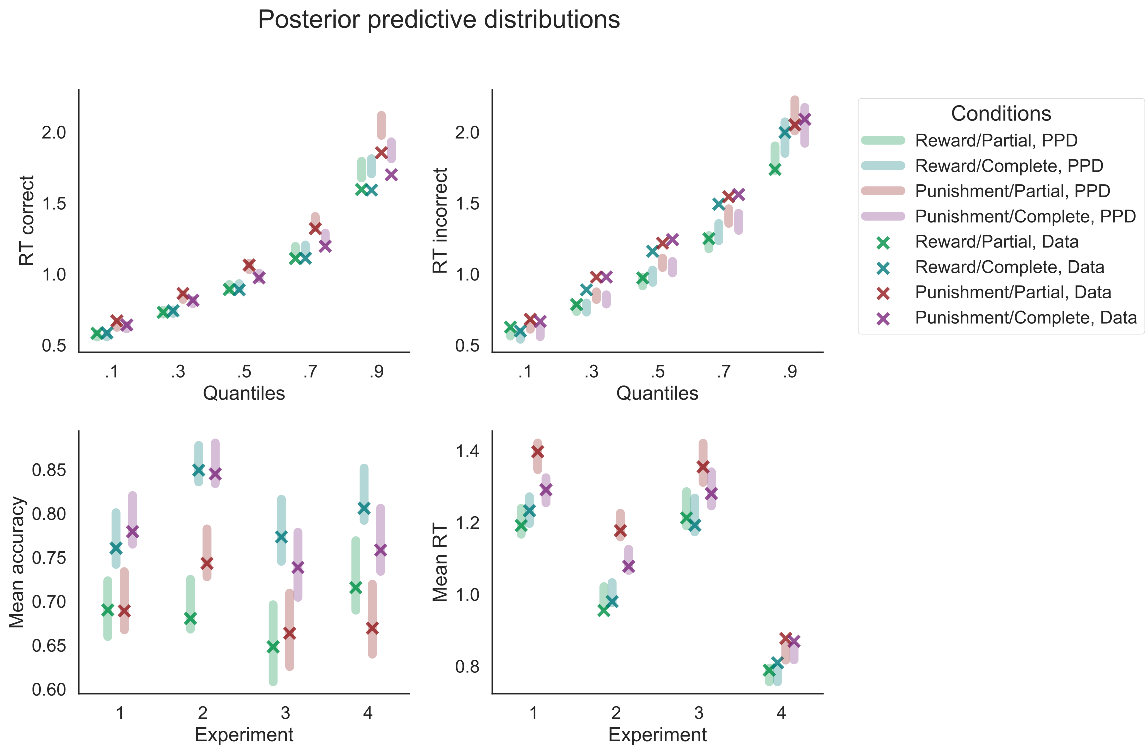

Figure B2. Posterior predictives of the hierarchical diffusion decision model. Posterior predictive distributions for mean accuracy (bottom-left), mean RT (bottom-right), RT quantiles of correct (top-left) and incorrect (top-right) responses. To assess how well the model fits the observed behavioral patterns, these measures were separately calculated across experiments and experimental conditions. The shaded areas represent the 95% Bayesian Credible Intervals, while the crosses represent the summary of the data.

Appendix C
Diffusion decision model parameter recovery

We performed parameter recovery of the Bayesian hierarchical diffusion decision model(DDM) used in the main analyses of this study. We generated data for four experiments using a simple DDM (with no across-trial variability), with the same number of participants and trials as in our study.

The generating group parameters (Table C1) were selected in order to generate a similar performance to the one observed across the experiments (Figure C1). Participants' parameters were sampled from the group distributions and NDT and threshold intercepts were lowered in Experiment 4 of .4 and .5, respectively.

We fitted the DDM following the same procedure used to fit the real data collected in the four experiments, as described in the Methods Section. To assess the quality of parameter recovery, we plotted the generating parameter values against a summary (mean and mode) of the estimated posterior distributions of the 89 participants (Figure C2). In general, all group parameters were well recovered, although for some parameters we observe a shrinkage towards the group mean (e.g., for the interaction coefficient of the threshold) which is a typical feature of hierarchical models. Individual drift-rate parameters estimates were also more spread compared to the generating ones.

Table C1
Generating parameters.

	Drift-rate	Threshold	NDT
Mean intercept	.6	.4	-.3
SD intercept	.2	.1	.1
Mean coefficients (valence, feedback, interaction)	.0, .5, .0	.20, .10, -.12	.12, .00, .10
SD coefficients (valence, feedback, interaction)	.10, .05, .10	.05, .05, .03	.04, .10, .08

Note. The generating parameters at the dataset level were used for the parameter recovery.

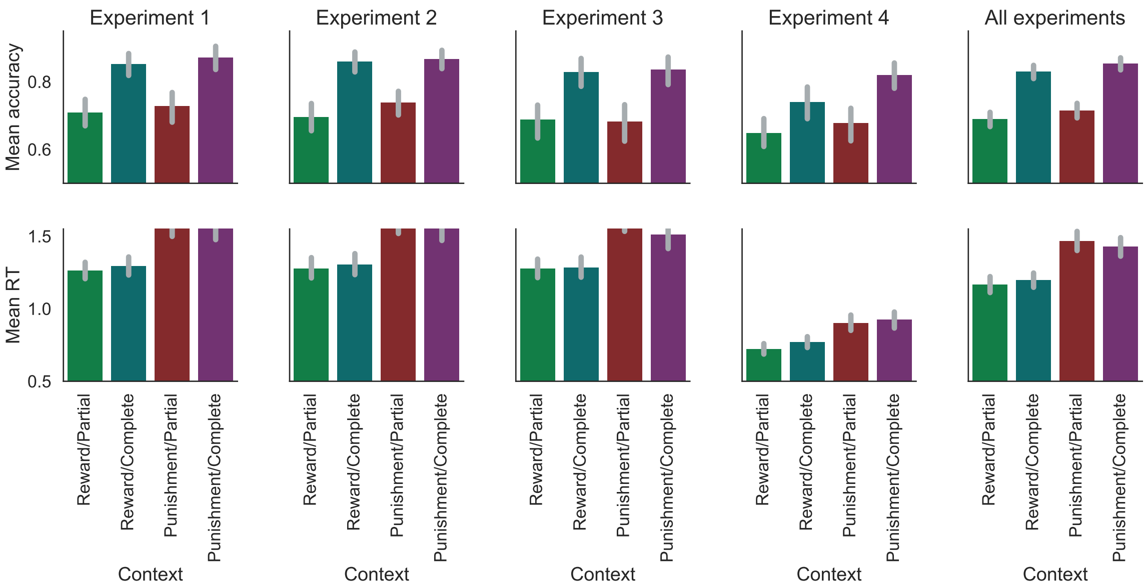

Figure C1. Simulated data. Mean accuracy and response times (RTs) of the simulated data, separately by experiment and by context.

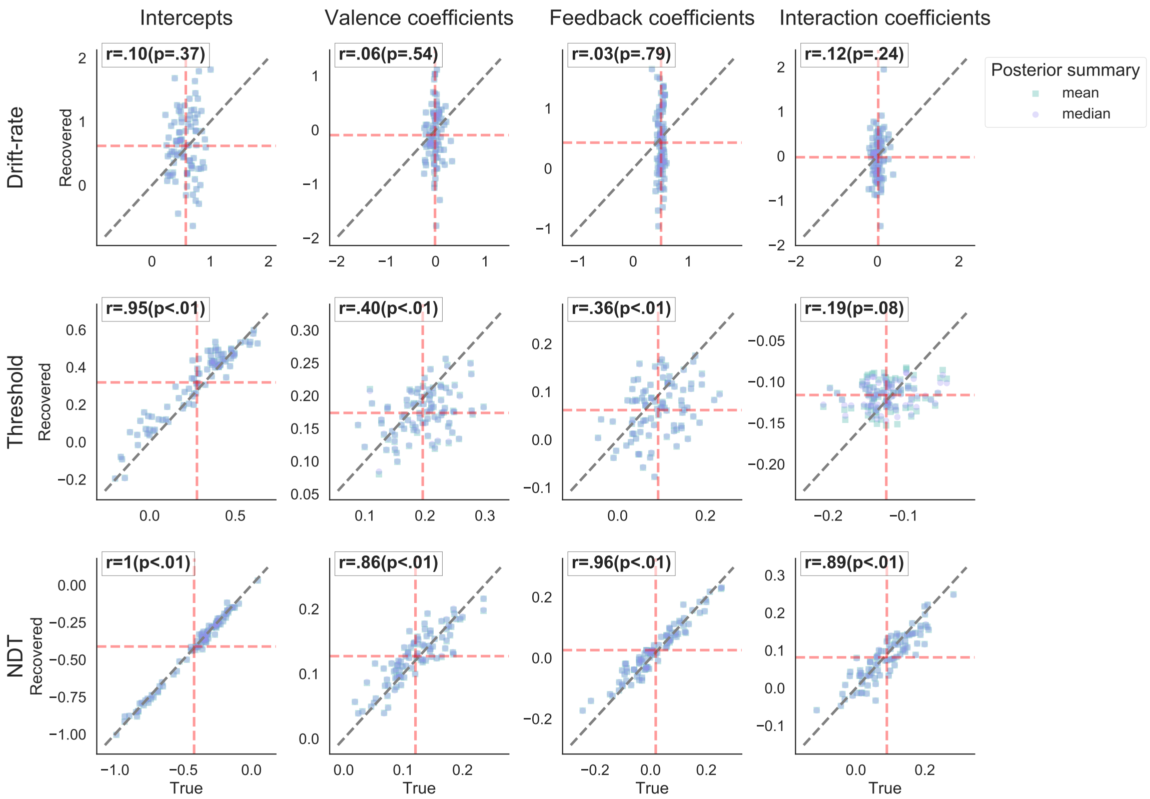

Figure C2. *True against recovered diffusion decision model individual parameters.* The dotted grey lines represent the identity lines, while the red dotted lines are the group mean parameters. We also calculated correlations between the true and the mean recovered individual parameters, indicated by the Pearson's ρ statistics.

Appendix D
Reinforcement learning model analyses

In this section, we report some details about the reinforcement learning modelling procedure.

The learning-rate parameters were given the following prior distributions:

$$\mu_\alpha \sim \mathcal{N}(.8, .5)$$
$$\sigma_\alpha \sim \mathcal{HN}(0, .5)$$
$$\alpha \sim \phi(\mathcal{N}(\mu_\alpha, \sigma_\alpha))$$

where μ_α is the group-level mean, σ_α is the group-level standard deviation, and α is the individual learning-rate. $\mathcal{N}$ is the normal distribution (with parameters mean and standard deviation), $\mathcal{HN}$ is the half-normal distribution, and ϕ is the cumulative density function of the standard normal distribution, transforming α so that $0 \leq \alpha \leq 1$. The decision parameters were given the following priors:

$$\mu_{v_{\text{coeff}}} \sim \mathcal{N}(0, 5)$$
$$\sigma_{v_{\text{coeff}}} \sim \mathcal{HN}(0, 3)$$
$$v_{coeff} \sim exp(\mathcal{N}(\mu_{v_{\text{coeff}}}, \sigma_{v_{\text{coeff}}}))$$

$$\mu_{a_{\text{int}}} \sim \mathcal{N}(0, 1)$$
$$\sigma_{a_{\text{int}}} \sim \mathcal{HN}(0, 1)$$
$$a_{\text{int}} \sim exp(\mathcal{N}(\mu_{a_{\text{int}}}, \sigma_{a_{\text{int}}}))$$

$$\mu_{a_{\text{coeff}}} \sim \mathcal{N}(0, .8)$$
$$\sigma_{a_{\text{coeff}}} \sim \mathcal{HN}(0, .5)$$
$$a_{\text{coeff}} \sim \phi(\mathcal{N}(\mu_{a_{\text{coeff}}}, \sigma_{a_{\text{coeff}}}))$$

$$\mu_{NDT_{\text{int}}} \sim \mathcal{N}(-1, 1)$$
$$\sigma_{NDT_{\text{int}}} \sim \mathcal{HN}(0, 1)$$
$$NDT_{\text{int}} \sim \mathcal{N}(\mu_{NDT_{\text{int}}}, \sigma_{NDT_{\text{int}}})$$

$$\mu_{NDT_{\text{coeff}}} \sim \mathcal{N}(0, 1)$$
$$\sigma_{NDT_{\text{coeff}}} \sim \mathcal{HN}(0, 1)$$
$$NDT_{\text{coeff}} \sim \mathcal{N}(\mu_{NDT_{\text{coeff}}}, \sigma_{NDT_{\text{coeff}}})$$

While the threshold could not be negative (because of the specific parameterization of Equation 8 and because a_{int} was exponentially transformed) to not allow the non-decision time to be negative, it was exponentially transformed at a trial level.

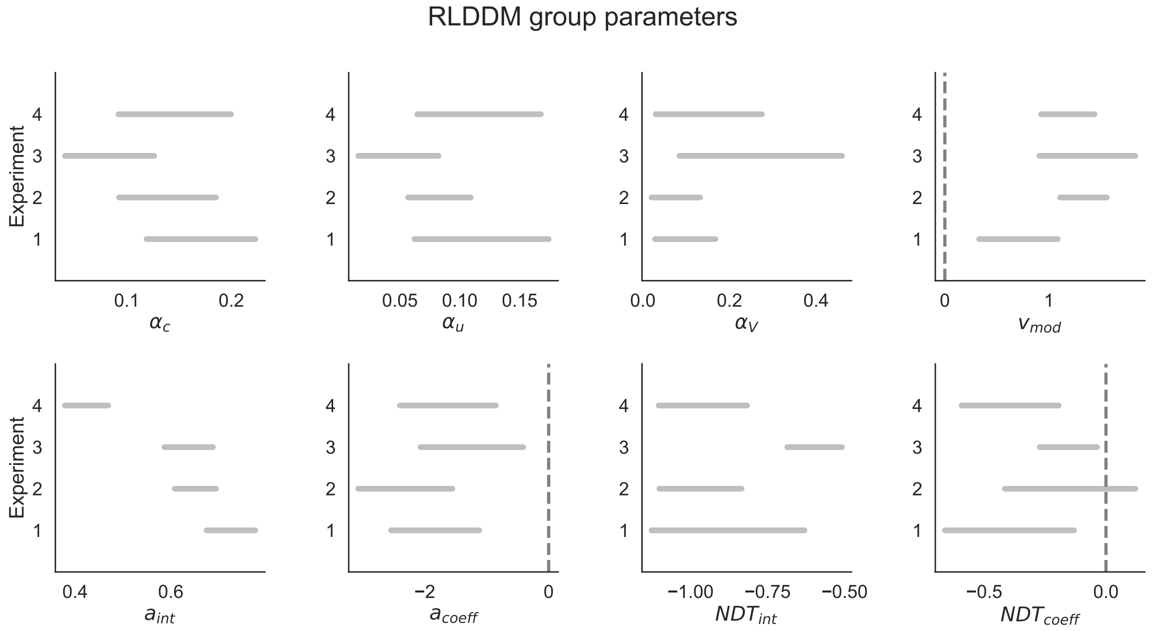

Figure D1. *95% Bayesian Credible Intervals of the posterior distributions of the hierarchical reinforcement learning diffusion decision model (RLDDM).* The posterior distributions of the RLDDM parameters at the group level (grey lines) are plotted separately by experiment.

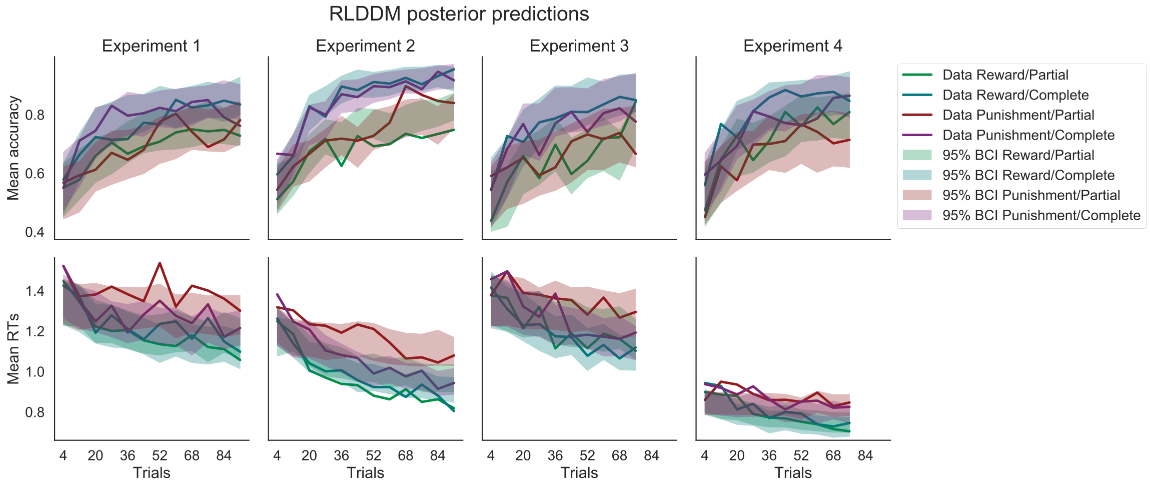

Figure D2. *Posterior predictives of the hierarchical reinforcement learning diffusion decision model (RLDDM).* Posterior predictives for mean accuracy (top row) and mean RTs (bottom row) in binned trials, separately for learning contexts. Each bin corresponds to 12 trials, which means 3 trials per choice context. Mean accuracy was calculated separately across experiments, contexts, and bins. The shaded areas represent the 95% Bayesian Credible Interval of the posterior predictive distributions. The hard lines represent the mean data.
